# Granulomatosis with Polyangiitis as a Cause of Sudden-Onset Bilateral Sensorineural Hearing Loss: Case Report and Recommendations for Initial Assessment

**DOI:** 10.1155/2021/6632344

**Published:** 2021-04-20

**Authors:** Paul R. Ratmeyer, Benjamin R. Johnson, Luis P. Roldan, Tania L. Kraai

**Affiliations:** University of New Mexico School of Medicine, Department of Surgery: Division of Otolaryngology and Head and Neck Surgery, Albuquerque, NM 87106, USA

## Abstract

Granulomatosis with polyangiitis (GPA) is a severe systemic vasculitis that commonly affects the paranasal sinuses, upper and lower respiratory tracts, and kidneys. GPA has also been associated with sensorineural hearing loss (SNHL), through inflammation of the cochlear apparatus. Early recognition, diagnostic laboratory evaluation, and appropriate treatment are essential to improve outcomes and achieve remission for patients with GPA. Here, we present a case of bilateral sudden sensorineural hearing loss (SSNHL) and distal symmetric polyneuropathy as the first presenting signs of GPA. A specific diagnostic work-up to rule out autoimmune inner-ear disease in patients with bilateral SSNHL is not clearly stated in the clinical practice guidelines from the American Academy of Otolaryngology-Head and Neck Surgery. The aim of this paper is to delineate an appropriate diagnostic work-up for patients with bilateral SSNHL when there is concern for autoimmune disease.

## 1. Introduction

In the United States, the prevalence of sudden sensorineural hearing loss (SSNHL) is 5 to 20 per 100,000 with an incidence of 4,000 cases per year. [1] Bilateral SSNHL, a specific subcategory, is an extremely rare condition accounting for less than 5% of cases with systemic disease typically implicated. New guidelines from the American Academy of Otolaryngology–Head and Neck Surgery (AAO-HNS) emphasize the need to delineate unilateral and bilateral SSNHL due to largely divergent etiologies [2]. Therefore, it has been proposed that bilateral SSNHL constitutes a medical emergency that warrants prompt and targeted investigation to exclude treatable and life-threatening causes [3].

Granulomatosis with polyangiitis (GPA) frequently manifests with hearing loss in addition to sinusitis, respiratory tract involvement, and intrinsic renal disease. Of note, HL may, at times, be the initial symptom [4]. Takagi et al. describe five separate otologic patterns in GPA: serous otitis media, chronic otitis media, sensorineural hearing loss (SNHL), vertigo, and facial nerve palsy [5]. Conductive hearing loss secondary to serous otitis media is the most common otologic presentation, but recent studies show that SNHL may be present in up to 47% of patients with GPA [6].

In addition, GPA constitutes only one of many systemic etiologies causing bilateral SSNHL outlined by the AAO-HNS [2] and better understanding may allow more complete assessment, diagnosis, and treatment of similar presentations. Furthermore, while practice guidelines present possible etiologies for bilateral SSNHL, to this point there is not a standardized diagnostic work-up with systemic autoimmune disease identification at the forefront. To this end, we present a case of GPA with profound bilateral SSNHL and distal symmetric polyneuropathy, highlighting systemic disease as a cause of bilateral SSNHL, and offer a suggested work-up for presenting patients.

## 2. Case Report

A 72-year-old female with seropositive rheumatoid arthritis, hypertension, and hypothyroidism presented with sudden-onset hearing loss with progressive weakness and paresthesias in a “stocking-glove” distribution. The patient reported fatigue, malaise, and recent unintentional weight loss which were previously attributed to an unresolved viral upper respiratory infection. She was transferred to our facility from an outpatient setting for evaluation of acute neurological symptoms.

**Figure 1:**
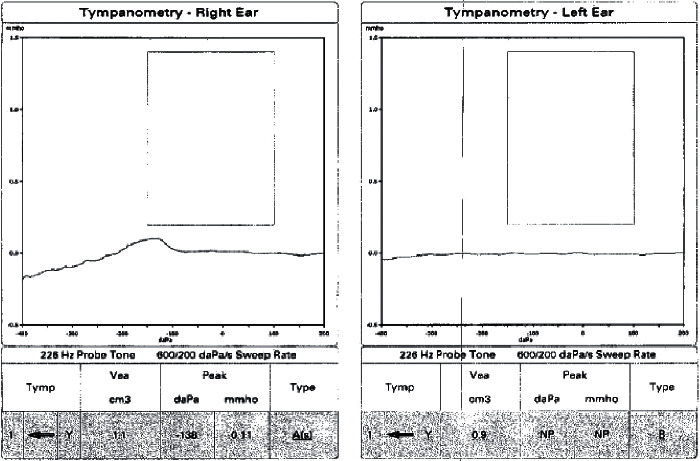
Type As and type B tympanograms in the right and left ear, respectively, upon admission.

**Figure 2:**
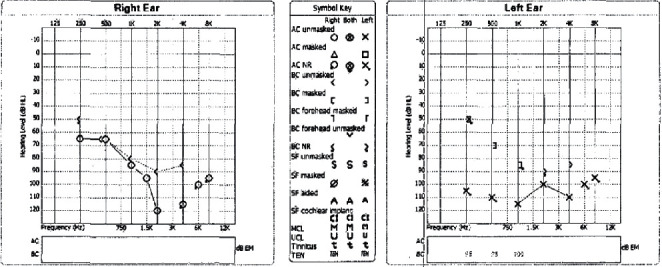
Audiogram at presentation showing profound bilateral hearing loss.

**Figure 3:**
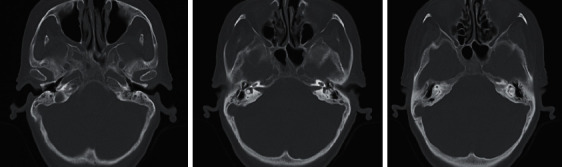
Sequential axial CT layers showing sclerotic mastoid air cells and aerated middle ears bilaterally with no evidence of effusion.

**Figure 4:**
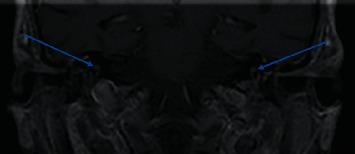
Coronal T1 spin echo showing abnormal bilateral postcontrast enhancement within the vestibule and semicircular canals.

**Figure 5:**
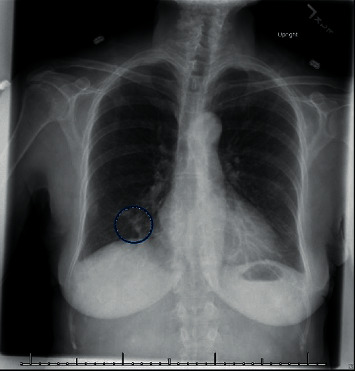
PA chest radiograph showing confluent opacity in the right lower lung.

During initial evaluation, the patient's hearing loss rendered her unable to discriminate any verbal stimuli. She was alert with a nonfocal neurological exam. Otomicroscopy revealed intact tympanic membranes with no apparent middle ear effusions; however, impedance audiometry suggested possible middle ear pathology with a right type A tympanogram and left type B (Figure 1). Pure tone audiogram revealed severe to profound right sensorineural hearing loss and profound left, possibly mixed hearing loss (Figure 2). Reliability of speech discrimination testing was questioned by audiologists due to patient responses (repeating the same word over and over despite appearing to understand instruction). Noncontrast CT was unremarkable for signs of acute hemorrhage or ischemia. Middle ear spaces appeared aerated bilaterally without evidence of effusion. The mastoid processes were sclerotic and hypopneumatized (Figure 3). T1 spin echo with gadolinium contrast (Figure 4) revealed increased signal intensity within labyrinths bilaterally suggesting an inflammatory process and breakdown of the blood-labyrinth barrier within the inner ear. Chest X-ray showed a right lower lobe opacity (Figure 5), which was unchanged from an X-ray conducted several months prior, suggesting a chronic pulmonary process.

On admission, loop diuretic ototoxicity and other systemic causes for bilateral SSNHL were considered. Additional studies revealed an elevated ESR >100 and strongly positive C-ANCA (1 : 640). The patient was given a preliminary diagnosis of GPA on day 5 of hospitalization. The diagnosis was confirmed with a kidney biopsy demonstrating early stage crescentic glomerulonephritis. Treatment of high-dose IV glucocorticoids and cyclophosphamide was initiated. One month following therapy, audiometry showed persistent, profound bilateral SSNHL with minimal interval improvement. Speech recognition scores remained unattainable with speech awareness thresholds of approximately 70 dB.

## 3. Discussion

Up to 70–100% of patients with GPA experience head and neck symptoms [7]. Otologic symptoms occur in a significant proportion of these patients and may manifest as conductive, mixed, or SNHL [5, 6]. Santos et al. describe the histopathologic changes potentially responsible for SNHL in GPA, including a vasculitic pattern of capillary occlusion, thickened vessel walls, and hemorrhage within the stria vascularis, spiral ligaments, and vestibule [8]. A similar pattern of small vessel inflammation is also present in other organs affected by GPA [9].

Hearing loss secondary to inflammatory disease was first described by McCabe in 1979, after observation of SNHL improvement following immune suppression. In his original description, McCabe prescribed high-dose steroids and cyclophosphamide for patients experiencing rapid onset and often bilateral hearing loss when clinical or laboratory evaluation failed to reveal an identifiable etiology [10]. In response to these findings, McCabe attempted to develop a series of screening tests for what he termed autoimmune inner-ear disease (AIED) [11]. In the decades that followed, attempts have been made to better define the pathogenesis and develop laboratory tests [12]; however, serologic testing is inconsistent often resulting in empiric treatment. Additionally, systematic review of the literature fails to show that these patients clearly benefit from steroids, immunosuppressive agents, or a combination of both [13].

Identifying causes of inner-ear disease presents a challenging clinical scenario. In the present case, early laboratory assessment with an autoimmune serum panel aided in the diagnosis of GPA. Early detection and treatment are particularly important as the mean survival of untreated generalized GPA is only 5 months, and nearly 90% of patients with GPA achieve remission with therapy [9].

In cases of bilateral SSNHL, current guidelines by the American Academy of Otolaryngology-Head and Neck Surgery suggest consideration of autoimmune, vascular, metabolic, infectious, neoplastic, toxic, traumatic, and inflammatory disorders. The guidelines include a broad differential for causes of bilateral SSNHL but offer no specific imaging or serologic recommendations to evaluate these patients [1]. Conversely, Schreiber et al. provide a more comprehensive assessment algorithm for initial evaluation of bilateral SSNHL [14]. Their proposed guidelines include urgent formal audiometry, clinical assessment, and MRI of the brain and internal acoustic meatus. Additionally, they suggest early laboratory assessment including CBC, ESR, CRP, ANA, anticardiolipin antibodies, lupus anticoagulant, anti-neutrophil cytoplasmic antibodies, clotting factors, and syphilis serology. Laboratory investigation for systemic diseases is supported by literature review finding of only 6/103 (5.8%) cases of bilateral SSNHL of idiopathic etiology while 17/103 (16.5%) were associated with an underlying autoimmune condition [3]. Distinct autoimmune diseases including GPA, while themselves separate, share significant rates of morbidity and mortality when left undiagnosed and untreated.

This case report highlights bilateral SSNHL as a possible indicator of systemic disease and the utility of a comprehensive and standardized work-up to exclude potentially life-threatening and treatable causes. Furthermore, idiopathic bilateral SSNHL should be considered a diagnosis of exclusion and rendered only after appropriate initial evaluation. Based on our experience with this case and review of the literature, patients presenting with bilateral SSNHL of unknown etiology should have the following imaging and laboratory studies to exclude autoimmune disease: CBC, ESR, CRP, ANA, anticardiolipin antibodies, lupus anticoagulant, rheumatoid factor, anti-neutrophil cytoplasmic antibodies, clotting factors, and urgent MRI of the brain and internal acoustic meatus.

## 4. Conclusion

GPA should be considered as a potential cause of bilateral SSNHL, especially in patients with vague constitutional symptoms, unexplained concomitant peripheral neuropathy, and laboratory findings suggestive of a chronic inflammatory state. This case highlights the importance of a comprehensive evaluation for systemic disease in bilateral SSNHL, including autoimmune, vascular, neoplastic and toxic etiologies.

## Data Availability

Data for this single patient case are available in the University of New Mexico electronic health records. Anonymized aspects of this case are available upon request to the corresponding author.
